# Metastatic small‐cell carcinoma of the bladder that maintains a complete response to chemoradiation therapy

**DOI:** 10.1002/iju5.12547

**Published:** 2022-10-19

**Authors:** Shinya Miyazaki, Takashi Ueda, Ryosuke Tamai, Akihisa Ueno, Terukazu Nakamura

**Affiliations:** ^1^ Department of Urology Saiseikai Suita Hospital Suita Osaka Japan; ^2^ Department of Urology Kyoto Prefectural University of Medicine Kyoto City Japan

**Keywords:** carboplatin, irinotecan, radiation, small cell carcinoma, urinary bladder

## Abstract

**Introduction:**

Small‐cell carcinoma of the urinary bladder has a poor prognosis, and no standard treatment has been established. We encountered a case of a patient with metastasis in which complete response and long‐term survival were obtained by treating the primary lesion with a combination of irinotecan, carboplatin chemotherapy, and radiation therapy.

**Case presentation:**

An 83‐year‐old man was diagnosed with a bladder tumor with liver metastasis. Small‐cell carcinoma was diagnosed via transurethral resection. Second‐line chemotherapy with irinotecan and carboplatin and irradiation of the primary lesion were significantly effective. The imaging evaluation showed a complete response. The therapeutic effect was maintained for 1 year, even after the discontinuation of chemotherapy.

**Conclusion:**

Irinotecan and carboplatin should be considered for the treatment of small‐cell carcinoma of the bladder. Irradiation of the primary lesion may also be useful if the extent of metastasis is low.

**Keynote message**

The prognosis of metastatic small‐cell carcinoma of the urinary bladder is extremely poor. However, multimodal treatment, as in this case, may contribute to improving the prognosis, even in older patients.

**Abbreviations & Acronyms**
CR: complete response
CT: computed tomography
EC: etoposide, carboplatin
EP: etoposide, cisplatin
IrC: irinotecan, carboplatin
IrP: irinotecan, cisplatin
MRI: magnetic resonance imaging
NCCN: National Comprehensive Cancer Network
NSE: neuron‐specific enolase
OS: overall survival
Pro‐GRP: pro‐gastrin releasing peptide
SCCB: small‐cell carcinoma of the urinary bladder
SCLC: small‐cell lung cancer

## Introduction

SCCB is extremely rare, accounting for less than 0.7% of all urinary bladder cancers.^1^ Its prognosis is relatively poor.^2^ The standard treatment for this condition has not yet been established owing to the small number of cases. Considering the similarities with SCLC, the NCCN guidelines recommend using carboplatin, etoposide, atezolizumab, or durvalumab for patients with advanced SCCB.^3^, ^4^ However, in the treatment of advanced SCLC, the use of irinotecan has been reported to significantly prolong the median OS compared with the use of etoposide.^5^ Here, we report a case of a patient with metastatic SCCB who survived for more than 2 years with CR following IrC therapy, and irradiation therapy of the primary lesion.

## Case presentation

**Fig. 1:**
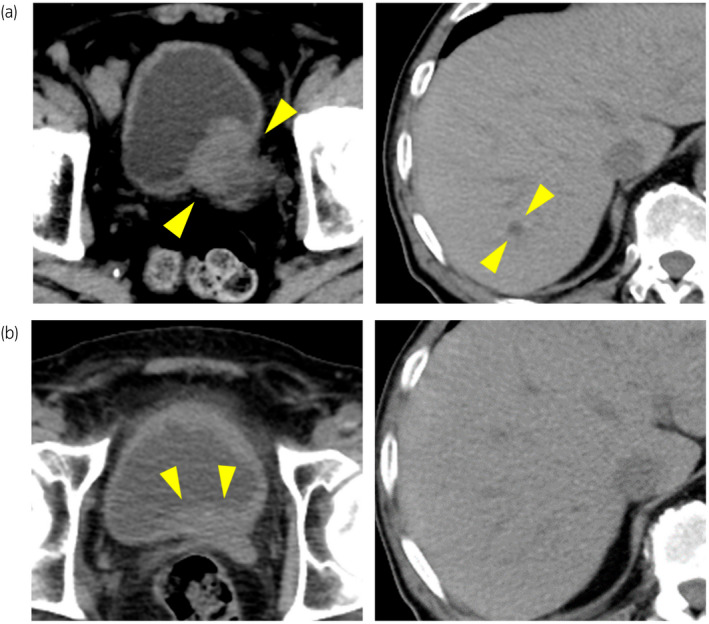
(a) CT at the first visit. A 43‐mm‐sized mass was detected on the left posterior wall of the bladder, accompanied by a twitch in the wall, and an 8‐mm‐sized low‐concentration nodule in size in the S6 region of the liver. (b) CT after irinotecan and carboplatin treatment. The bladder tumor shrank significantly, with only a mild thickening of the bladder wall. The liver tumor was obscured.

**Fig. 2:**
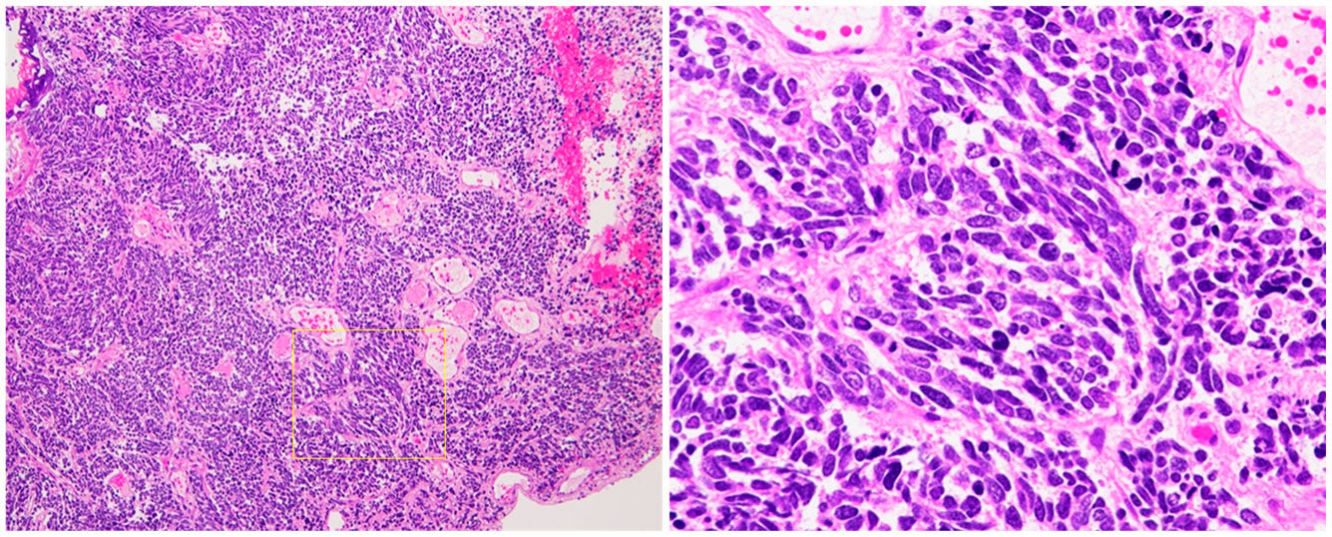
The pathological tissue sampled by transurethral resection. Microscopic images of hematoxylin and eosin staining of the specimens are presented in the panels, and 40× and 200× fields of view. Poorly differentiated tumors consisting of solid proliferations of round‐ to spindle‐shaped cells were observed. The nuclear chromatin in the cytoplasm of each tumor cell appeared fine and poor. The adjacent cells often contact each other, similar to a mold. Urothelial carcinomas were not observed.

**Fig. 3:**
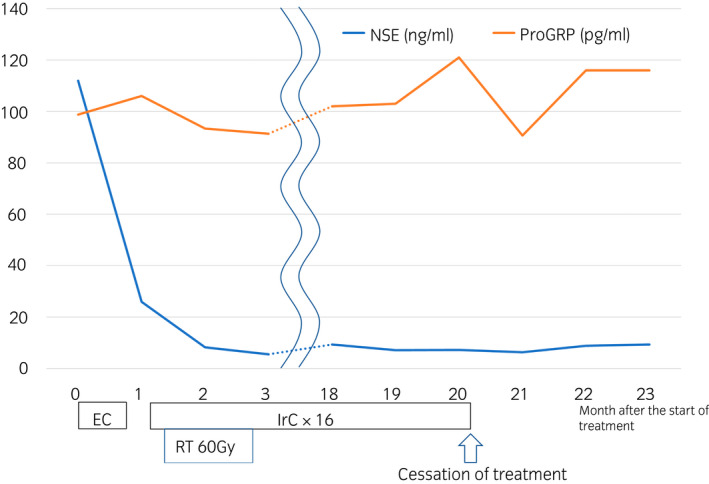
Treatment course and changes in tumor markers. The NSE levels returned to normal after one course of IrC therapy. Even after the completion of treatment, the NSE levels did not increase.

An 83‐year‐old man visited our institution with complaints of gross hematuria. CT and MRI revealed a 43‐mm‐sized muscular invasive tumor on the left side of the bladder wall, left‐side hydronephrosis, and 10‐mm‐sized liver metastasis (Fig. 1a). A non‐papillary tumor was found on cystoscopy, but the left ureteral orifice could not be confirmed. The NSE and Pro‐GRP levels increased to 112 ng/mL and 98.8 pg/mL, respectively. Moreover, the serum creatinine level increased from 1.31 to 2.71 mg/dL; hence, percutaneous left nephrostomy was performed. Transurethral resection of the bladder tumor was performed, and the patient was pathologically diagnosed with SCCB with muscular layer infiltration (Fig. 2). The nodule in the liver was hypoechoic in the surrounding area and hyperechoic on the inside, which was consistent with metastasis. Based on the imaging findings, the patient had T3bN0M1. Due to prolonged renal dysfunction, EC were selected as primary systemic chemotherapy for SCCB. After 1 cycle of chemotherapy, the NSE level decreased to 25.9 ng/mL, while the Pro‐GRP level increased to 106 pg/mL. The CT scan showed an increase in bladder tumor size from 33 to 51 mm. The treatment was considered unsuccessful; hence, irinotecan therapy was selected as second‐line chemotherapy. Owing to the patient's age, surgery was considered unsuitable, and radiation therapy of the primary lesion was performed (60 Gy/30 fractions) for local control and prevention of hematuria. After 2 cycles of IrC therapy, a repeat CT scan showed shrinkage of the bladder tumor and liver metastasis (Fig. 1b). IrC therapy was discontinued due to the occurrence of bone marrow suppression after 16 cycles (Fig. 3). One year after the treatment, no signs of tumor recurrence were detected on urinary cytopathology, cystoscopy, CT, or MRI.

## Discussion

This case suggests that IrC therapy may be useful as second‐line systemic chemotherapy for SCCB. In those with only a few metastases, such as our patient, irradiation of the primary lesion may be considered combined with systemic chemotherapy.

Considering the similarities with SCLC, as a treatment for metastatic SCCB, the NCCN guidelines recommend the use of carboplatin, etoposide, atezolizumab, or durvalumab as first‐line treatment.^3^, ^4^ In Japan, IrP therapy is also recommended as it significantly prolongs the OS compared with EP therapy in a previous randomized controlled trial on metastatic SCLC.^5^ A previous retrospective study examined the therapeutic factors of SCCB and reported that cisplatin‐based chemotherapy was an independent factor.^6^

The effectiveness of irinotecan‐based chemotherapy has also been reported in metastatic extrapulmonary neuroendocrine tumors, including small‐cell carcinoma. The study examined 34 patients, including one with a primary lesion in the bladder, who showed progression after first‐line treatment with platinum‐based chemotherapy and used irinotecan as second‐line treatment. Six patients achieved partial response (17.6%), and nine patients showed stable disease (26.5%).^7^ Another study reported that IrP therapy was selected for neoadjuvant chemotherapy for small‐cell carcinoma in the ureter. In that study, the small‐cell carcinoma component disappeared from the resected specimen.^8^

In this case, radiation therapy and chemotherapy were administered to the bladder to prevent bleeding. Several reports have examined the efficacy of radiation therapy alone and in combination with chemotherapy. Chau *et al*. retrospectively examined the treatment of localized SCCB in 200 patients.^9^ A total of 61 patients underwent radical cystectomy, while 104 received radiation therapy. No significant difference was observed in the median OS between patients who underwent radical cystectomy and those who received radiation therapy. The median OS times were 26.7 months in the radical cystectomy group and 30.0 months in the radiation therapy group. Akamatsu *et al*. retrospectively examined 12 patients with SCCB who underwent definitive radiation therapy.^10^ In eight of the 12 patients who received systemic chemotherapy, the 3‐ and 5‐year survival rates were 50% and 33.3%, respectively, while the 3‐ and 5‐year local control rates were 66.7% and 55.6%, respectively. The OS and progression‐free survival rates were significantly improved in the systemic chemotherapy combination group.

In this case, the Pro‐GRP level remained slightly elevated. The Pro‐GRP level tends to increase in patients with chronic renal failure.^11^ Chronic renal failure was also prolonged in this case, which was thought to cause high Pro‐GRP levels. NSE has been reported as a useful marker for diagnosing and treating SCLC,^12^ and its value decreases on positive response to chemotherapy. In addition, it remains low even after the completion of chemotherapy, reflecting disease activity.

In our patient, the CR was maintained for a long period even after the end of systemic chemotherapy, thus extending the prognosis. Ogihara *et al*. retrospectively examined 128 patients who experienced metastasis after total cystectomy. The 2‐year cancer‐specific survival rate was significantly higher in the group that met the following criteria for oligometastasis: solitary metastatic organ, ≤3 metastatic lesions, the largest diameter of metastatic foci of ≤5 cm, and no liver metastasis. Multivariate analysis showed that the absence of chemotherapy or resection of metastases was an independent risk factor for cancer‐specific death.^13^ In this case, the metastatic lesion developed in the liver; although it did not meet the above study criteria, the size of the metastatic lesion was relatively small, and long‐term chemotherapy was considered to have contributed to the prolonged prognosis.

Combining chemotherapy and irradiation of the primary lesion for SCCB with a small amount of metastasis may improve the prognosis; however, further investigation is required to verify this finding.

## Author contributions

Shinya Miyazaki: Writing – original draft. Takashi Ueda: Writing – review and editing. Ryosuke Tamai: Data curation. Akihisa Ueno: Data curation. Terukazu Nakamura: Supervision.

## Conflict of interest

The authors declare no conflict of interest.

## Approval of the research protocol by an Institutional Reviewer Board

Not applicable.

## Informed consent

Consent was obtained from the patient for publication.

## Registry and the Registration No. of the study/trial

Not applicable.
